# Temporal and Spatial Distribution Characteristics of Crosstalk Lines Generated by Irradiating Progressive Scan Charge-Coupled Device Camera with Continuous Laser

**DOI:** 10.3390/s24123966

**Published:** 2024-06-19

**Authors:** Chenghao Yu, Jifei Ye, Hao Chang, Nanlei Li, Wei Guo

**Affiliations:** State Key Laboratory of Laser Propulsion & Application, Department of Aerospace and Technology, Space Engineering University, Beijing 101416, China; yuchenghao0536@163.com (C.Y.); changhao5976911@163.com (H.C.); 15910624233@163.com (N.L.); guoweikl@126.com (W.G.)

**Keywords:** continuous laser, laser irradiation, progressive scan CCD, interference effect, crosstalk line, spatial distribution

## Abstract

To study the interference effect of the laser in motion mode on a CCD, the continuous laser with the wavelength of 532 nm at different motion speeds was used to scan the CCD. The experimental results show that the crosstalk phenomenon produced by static and dynamic irradiation is significantly different. When the continuous laser statically radiates the CCD, the vertical crosstalk line is observed in the output image. The gray values of the crosstalk line are divided into two stages, with the increase of the laser fluence: linear increase and saturation, which correspond to different formation mechanisms of the crosstalk lines, respectively. In addition, when the irradiation duration of the static laser is less than the integration time of CCD, the effect of delay time on the spatial distribution of the crosstalk line is identified. In addition, when the laser irradiates the CCD at different scanning speeds, crosstalk lines with certain slopes are observed. The slope of the crosstalk line is determined by the scanning speed of the continuous laser and the integration time of the CCD. The results show that the delay time and the irradiation position have important effects on the spatial distribution of the laser spot and crosstalk lines.

## 1. Introduction

Charge coupled device (CCD), which consists of a metal oxide semiconductor (MOS), is one of the most common photodetectors [1]. It has the advantages of small size, low power consumption, high detection sensitivity and resolution, and large dynamic range [2]. Applications of CCD have been attracting increasing attention because they are providing better solutions for several increasingly important fields, such as medical diagnosis [3], industrial detection [4], and intelligent driving [5]. In recent years, the interaction between different photodetectors and light sources has gradually become a research hotspot [6,7,8,9]. As the semiconductor material in the component of CCD has strong absorption of laser energy, the CCD is susceptible to being interfered with or damaged by laser. When the laser irradiation stops, the performance of CCD could gradually recover, which is called laser interference or laser dazzling [10]. However, the decline in the performance of CCD or complete failure is called laser damage [11].

As the laser energy increases from little to large, laser interference on CCD occurs first [12]. In recent years, the laser interference generated by irradiating many kinds of CCD image sensors, including linear array CCD [10], time delay integration CCD (TDI-CCD) [13,14], frame transfer CCD (FT-CCD) [15], and interline transfer CCD (IT-CCD) [16], has been studied extensively. Moreover, saturation [15], various crosstalk [17], background fringes [13], side spots [10,14], spot tail [18,19], and excessive saturation [20] have been observed in the recorded output images of CCD. Furthermore, through theoretical analysis and numerical simulations, the saturation and crosstalk thresholds of CCD were achieved [21]. Based on the location where the abnormal responses occur, anomalous responses could be summarized as distortions in the electrical signal, charge distribution, and light distribution [22]. In addition, laser parameters have an important influence on the interference effect of CCD. The anti-interference ability of CCD to continuous laser with different wavelengths was investigated by the quantum efficiency of CCD material and penetrability to lasers: 532 nm > 632.8 nm [16]. The brightness in the view field of CCD to vibrate periodically was observed by using the repetitive picoseconds pulse laser, and quantitative expressions were given [13]. In contrast, in experiments of CCD irradiated by continuous laser and pulsed laser with different repetition rates, it is found that the interference effect caused by continuous laser is more intense [23]. In addition, the setting parameters of CCD also have an important influence on the laser interference effect. The influence of the integration time on the laser disturbing effect has been comparatively investigated. It is found that the pixel numbers of the different gray levels gradually increase with the extension of the integration time [24]. By comparing the laser spot image with the distribution of the gray scale under different gains, the increase of the CCD gain results in a larger number of saturated pixels and enhances the laser disturbing effect [25]. However, when the irradiation duration of the laser is less than the integration time of CCD, the interference effect on CCD irradiated by laser at different delay times is not clear [6,26,27]. In addition, the studies of laser interference on CCD have mainly used static laser as an irradiation source. Therefore, it is necessary to carry out interference studies on CCD irradiating by moving laser.

In this study, the experimental system for irradiating CCD with the continuous laser in static and motion modes was constructed. The crosstalk phenomenon of the progressive scan CCD by static and dynamic laser irradiation was observed in the output images of the CCD. In static irradiation, the gray values of the pixels on the crosstalk lines were extracted to study the effect of laser fluence. Furthermore, when the irradiation duration of the static laser was less than the integration time of the CCD, the spatial distribution of the crosstalk line was investigated at different delay times. In dynamic irradiation, the crosstalk phenomenon resulting from scanning the CCD with the continuous laser at different speeds was analyzed by adjusting the rotational speed of the motorized rotation stage. In addition, the typical irradiation positions at the bottom, middle, and top of the CCD were chosen to investigate the spatial distribution of crosstalk lines generated by scanning the CCD with continuous laser in motion mode.

## 2. Experimental Setup

**Figure 1:**
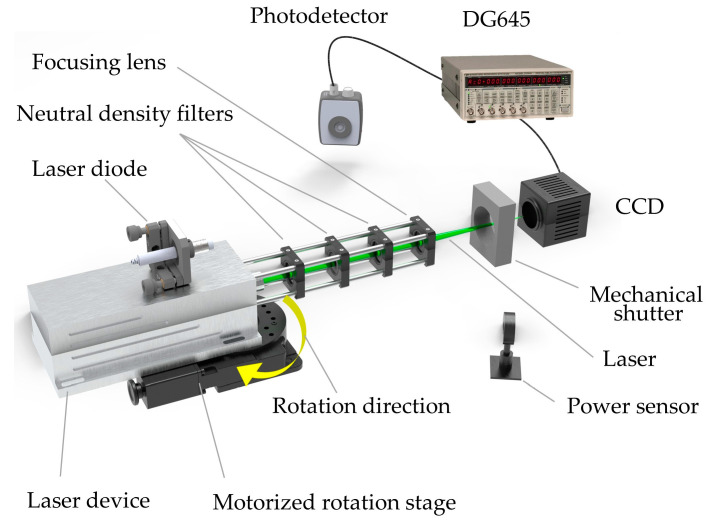
Schematic diagram of the experimental system for interfering with the CCD by the continuous laser in static and motion modes.

The schematic diagram of the experimental system is shown in Figure 1. The CCD camera (BM-141GE, JAI, Copenhagen, Denmark) employed in the experiment is equipped with a CCD image sensor (ICX285AL, SONY, Tokyo, Japan), which is a 2/3” progressive scan monochrome version. Number of effective pixels of the CCD image sensor is 1392 × 1040 and size of the pixel is 6.45 μm × 6.45 μm. Since the wavelength of the peak spectral response of the CCD is about 520 nm, the continuous laser with the wavelength of 532 nm (MGL-F-532 nm-2W, CNI, Changchun, China) was chosen as the laser source for irradiating the CCD. The laser reaches the surface of the CCD chip through a focusing lens with a focal length of 300 mm. The distance between the focusing lens and the surface of the CCD chip was 300 mm. The laser spot size on the surface of the CCD chip was measured by the high-resolution laser beam profiling system (LaserCam-HR II, Coherent, Saxonburg, PA, USA). The laser power reaching the CCD was adjusted by neutral density filters with transmittance of 50%, 10%, and 1%, respectively. To ensure the stability of the laser power, the power sensor (PD300R-3W, Ophir, Tokyo, Japan) was used to measure the laser power before each test. When the continuous laser statically irradiates the CCD, the laser optical axis is perpendicular to the surface of the CCD chip. The irradiation time of the continuous laser was controlled by the mechanical shutter (GCI-7103M, Daheng Optics, Beijing, China). In addition, the focusing lens and neutral density filters were mounted on the optical cage system. Furthermore, the optical cage system was firmly connected to the laser source equipment. To ensure that the continuous laser could scan the CCD at different moving speeds, the laser was fixed to the motorized rotation stage, whose rotation speed could be adjustable from 30°/s to 80°/s. Therefore, by controlling the rotation speed of the motorized rotation stage, the relative motion between the laser spot and the CCD can be realized. In addition, the focusing lens with the appropriate depth of field ensures that the laser spot remains basically unchanged when the laser scans the CCD. Temporal sequence control between the laser and the image acquisition of the CCD was achieved using a digital delay pulse generator (DG654, Stanford Research Systems, Sunnyvale, CA, USA). Since the laser energy attenuated by the neutral density filters was weak, the laser diode was fixed to the top of the laser as a reference light source to ensure the stability of the timing control. When the reference light source begins to rotate and first irradiates to the photodetector near the CCD, the photodetector generates a signal and triggers DG645 to achieve timing control. In this experiment, only one image was recorded when the CCD camera received an external trigger signal.

**Figure 2:**
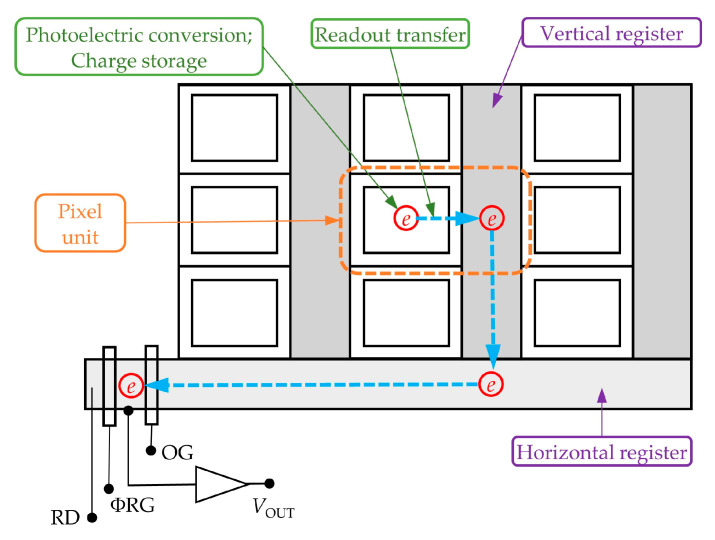
Construction diagram of the progressive scan CCD image sensor.

The structure diagram of the progressive scan CCD is shown in Figure 2. The CCD is mainly composed of photosensitive units, vertical registers, horizontal registers, and an amplifier [28]. When the laser is irradiated to the pixel, signal charges will be generated by the photosensitive area of the pixel and are collected in the collecting potential well. The process by which the collected signal charges are transferred from the collecting potential well to the transmission potential well in the vertical register is called the readout transfer. After the readout transfer occurs, the signal charges will be driven by the transfer clock along the vertical register until they reach the horizontal register. Subsequently, the signal charges, which are driven by the transfer clock of the horizontal register, will be transferred to the amplifier and finally restored to an output image through A/D conversion.

## 3. Results and Discussion

### 3.1. CCD Irradiating by Continuous Laser in Static Mode

#### 3.1.1. Effect of Laser Fluence

**Figure 3:**
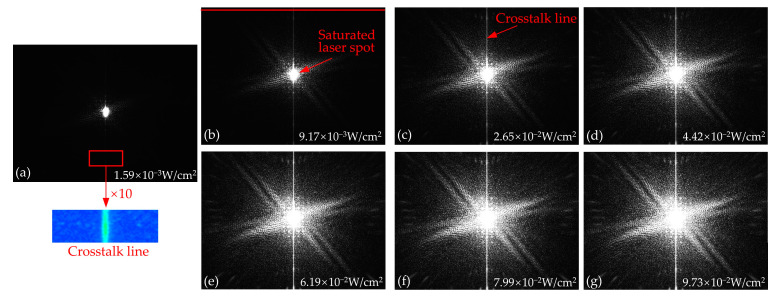
The output images generated by irradiating the progressive scan monochrome CCD with continuous laser in static mode at typical laser fluences. The integration time of the CCD was set to 32 ms. The 10× local magnification image has been processed with a false color for improved clarity in observation. The laser fluences of the continuous laser used to irradiating the CCD are (**a**) 1.59 × 10^−3^ W/cm^2^, (**b**) 9.17 × 10^−3^ W/cm^2^, (**c**) 2.65 × 10^−2^ W/cm^2^, (**d**) 4.42 × 10^−2^ W/cm^2^, (**e**) 6.19 × 10^−2^ W/cm^2^, (**f**) 7.99 × 10^−2^ W/cm^2^, and (**g**) 9.73 × 10^−2^ W/cm^2^, respectively.

Figure 3 shows the output images generated by irradiating the CCD with a continuous laser in the static mode at typical laser fluences. Each output image contains a saturated laser spot and vertical crosstalk line. During the process of laser irradiation on the photosensitive area of the CCD pixel, signal charges will be continuously generated. When the amount of signal charge exceeds the maximum capacity of the collecting potential well, signal charges will break through the barrier between the collection potential well and the transmission potential well and overflow from the collecting potential well to the transport potential well of the vertical register. Subsequently, the crosstalk phenomenon in output images occurs as the overflow charges spread further in the vertical register. In addition, because the laser fluence is concentrated on a few pixels at the center of the laser spot, the crosstalk line passes through the center of the laser spot. Furthermore, when the continuous laser at a wavelength of 532 nm irradiates the CCD, the threshold for generating crosstalk lines in the output image is about 1.59 × 10^−3^ W/cm^2,^ as shown in Figure 3a. In addition, with an increase in laser fluence, the divergence of light around the main spot is observed, which is caused by the diffraction effect of the diaphragm in the optical system [29]. In addition, the mesh distribution of the light points appears around the laser spot. The reason for the mesh distribution of the light points is due to the interference effect on the photosensitive surface produced by the reflective lights, which is caused by the reflection of the lens and detector [29].

**Figure 4:**
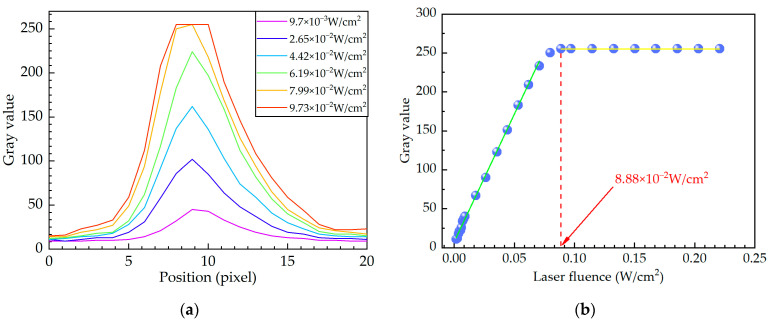
(**a**) The variation in gray values of pixels along the horizontal direction of the crosstalk line at typical laser fluences; (**b**) The variation in the peak gray value of the crosstalk line with the laser fluence.

The gray values of the crosstalk lines extracted along the red line at the top of Figure 3 vary with the laser fluence, as shown in Figure 4a. When the laser fluence is constant, the gray value is large in the middle and small on both sides, which is similar to the Gaussian distribution. In addition, as the laser fluence increases, the gray value of the crosstalk line gradually increases until it reaches a saturated value of 255, as shown in Figure 4a. To further investigate the influence of the laser fluence on the crosstalk lines, the variation in the peak gray value of the crosstalk line with the laser fluence is shown in Figure 4b. As shown in Figure 4b, the gray value first increases linearly with increasing laser fluence. However, when the laser fluence is greater than 8.88 × 10^−2^ W/cm^2^, the gray value remains constant at 255 as the laser fluence increases. As the photoelectric conversion is continuous during laser irradiation, the overflow of signal charges occurs continuously after the collecting potential well is full. When the laser fluence is less than 8.88 × 10^−2^ W/cm^2^, the transmission potential well of the vertical register does not reach its maximum capacity because the gray value of the crosstalk line in Figure 4b is not saturated. Therefore, each transmission potential well in the vertical register passing through the overflowing pixel will acquire an equal amount of signal charge, which eventually forms the crosstalk line with unsaturated gray values, as shown in Figure 3c. When the laser fluence is greater than 8.88 × 10^−2^ W/cm^2^, the amount of overflow charge reaches the maximum capacity of the transmission potential well in the vertical register, and the barrier between the adjacent transmission potential well loses its limiting effect. The excess signal charges will overflow from the transmission potential wells and drift sequentially from near to far into neighboring transmission potential wells, resulting in crosstalk lines with saturated gray values of pixels, as shown in Figure 3g.

#### 3.1.2. Effect of Delay Time

**Figure 5:**
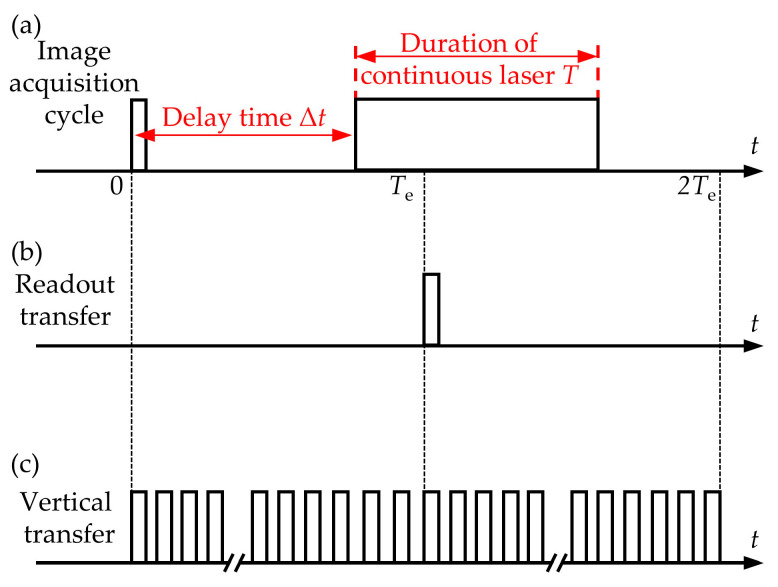
(**a**) Sequence diagram of image acquisition and laser loading; (**b**) Sequence diagram of readout transfer; (**c**) Sequence diagram of vertical transfer.

When the duration of laser irradiation is less than the integration time of the CCD, the effect of the delay time between the moment the laser is loaded and the moment the CCD begins integrating on the crosstalk lines is not clear. Therefore, the continuous laser was modulated by a mechanical shutter to reduce the duration of laser irradiation to less than the integration time of the CCD in this experiment. The integration time *T*_e_ of the CCD was set to 32 ms. *T*_e_/2 = 16 ms was selected as the typical duration of laser irradiation. The operating mode of the CCD was set to the single-frame mode. In single-frame mode, only one output image was captured in one test. As shown in Figure 5a, the delay time between the moment the laser starts loading and the moment the CCD starts integrating is defined as Δ*t*. When the laser starts irradiating the CCD after the CCD starts integration, Δ*t* > 0 is defined, and vice versa, Δ*t* < 0. The duration of the laser irradiation is defined as *T*. As shown in Figure 5b, the readout transfer action occurs at the end of the CCD integration. Moreover, for the progressive scan CCD in the single-frame mode, only one action of readout transfer occurs in one test. The signal in Figure 5c represents the movement of the transmission potential well carrying the signal charges in the vertical register from the pixel where it is located to the next pixel. In addition, the queue of transmission potential wells in the vertical register is in directional motion for the majority of the image acquisition period.

**Figure 6:**
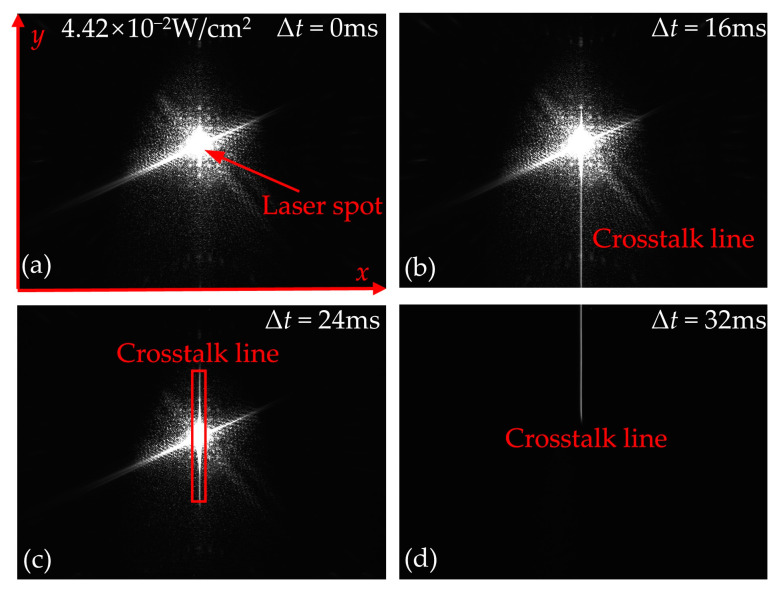
Output images produced by irradiating the CCD at delay times of (**a**) Δ*t* = 0 ms, (**b**) Δ*t* = 16 ms, (**c**) Δ*t* = 24 ms, and (**d**) Δ*t* = 32 ms, respectively. The integration time of the CCD was set to 32 ms, and the duration of the laser irradiation was set to 16 ms.

**Figure 7:**
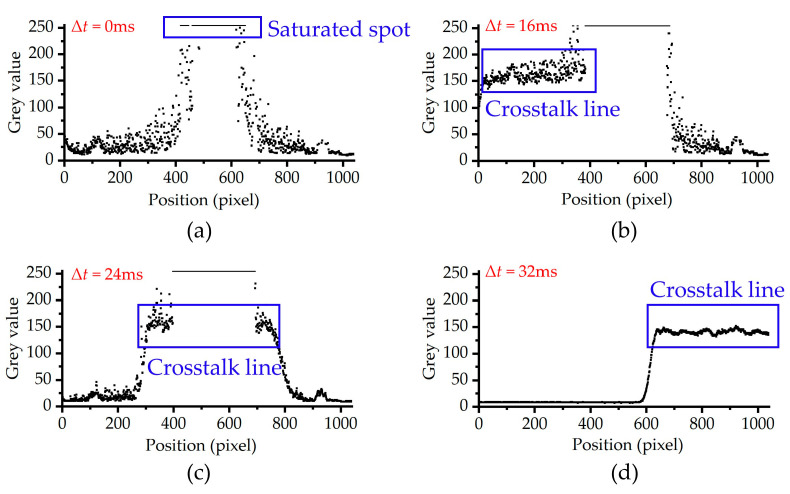
The trend of the gray value of the crosstalk lines along the *y*-direction at delay times of (**a**) Δ*t* = 0 ms, (**b**) Δ*t* = 16 ms, (**c**) Δ*t* = 24 ms, and (**d**) Δ*t* = 32 ms, respectively. The integration time of CCD was set to 32 ms, and the duration of laser irradiation was set to 16 ms.

When the irradiation duration of the static laser is less than the integration time of CCD, Figure 6 shows the output images of the CCD irradiated by the continuous laser at typical delay times. To observe the spatial distribution of crosstalk lines more clearly, Figure 7 shows the trend of the gray value of the crosstalk lines along the *y*-direction, which corresponds to Figure 6. As shown in Figure 6a and Figure 7a, when the delay time Δ*t* is 0 ms, only the laser spot is observed in the output image. As shown in Figure 6b and Figure 7b, when Δ*t* is 16 ms, in addition to the laser spot, the crosstalk line located below the laser spot appears in the output image. When Δ*t* is 24 ms, the output image contains the laser spot and the crosstalk line that is symmetric about the laser spot in Figure 6c and Figure 7c. When Δ*t* is 32 ms, only the crosstalk line located in the upper part of the image is observed, as shown in Figure 6d and Figure 7d. The experimental results in Figure 6 show that the delay time Δ*t* has an important effect on the spatial distribution of the crosstalk lines and laser spots. When the laser irradiation begins before the moment the readout transfer occurs, the signal charges will be generated and stored in the collecting potential wells. When the readout transfer occurs, the signal charges in the collecting potential well are transferred to the corresponding transmission potential well in the vertical register. Therefore, the final output image of the CCD contains the laser spot, as shown in Figure 6a–c. Since only one readout transfer is performed in one test, as shown in Figure 5b, when the laser irradiation begins after the moment the readout transfer occurs, the signal charges that are gathered at the collection potential wells cannot be transferred into the transmission potential wells by the readout transfer. Therefore, the laser spot could not be observed in the output image, as shown in Figure 6d and Figure 7d.

**Figure 8:**
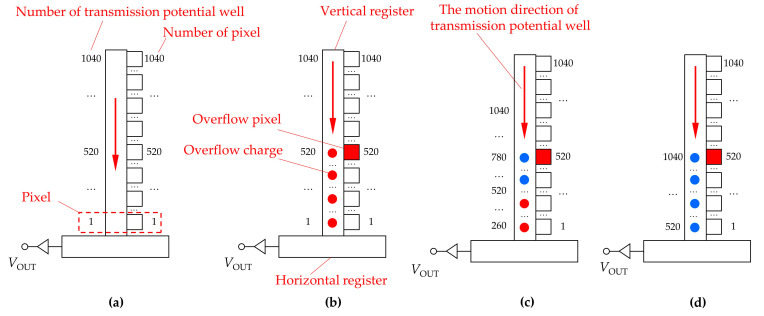
(**a**) Schematic diagram of the binding relationship between pixels and transmission potential wells at the time readout transfer occurs; (**b**) Schematic diagram of the spatial distribution of overflow charge at the end of laser irradiation when Δ*t* = 16 ms; (**c**) Schematic diagram of the spatial distribution of overflow charge at the end of laser irradiation when Δ*t* = 24 ms; (**d**) Schematic diagram of the spatial distribution of overflow charge at the end of laser irradiation when Δ*t* = 32 ms. The red and blue overflow charges are obtained by the transport potential well before and after the readout transfer, respectively.

As shown in Figure 8a, the transmission potential well in the vertical register will be bound to the pixel where it is located at the moment of readout transfer. After the readout transfer action, the transmission potential well will move toward the downstream. The pixels and transmission potential wells that are bound to each other are labeled with the same number in Figure 8. The red overflow charge shown in Figure 8 is obtained by the transport potential well before the readout transfer, and the blue overflow charge is obtained by the transport potential well after the readout transfer. The effect of the overflow charge on the transmission potential well will eventually be the effect on the pixel bound to the transfer potential well. When the delay time Δ*t* is 16 ms, the spatial distribution of the overflow charges at the end time of the laser irradiation is shown in Figure 8b. During laser irradiation, the transmission potential wells pass sequentially through the pixel where the charge overflow occurs and acquire the overflow charges. At the end of the laser irradiation, the overflow charges are transferred to the downstream pixel at positions No. 1 to No. 520. When the readout transfer action occurs, these charges in the transmission potential wells are considered to be obtained from the bound pixels, i.e., they are considered to be obtained from pixels No. 1 to No. 520. Therefore, the spatial distribution of the crosstalk line in Figure 6b is formed. Moreover, when Δ*t* is 24 ms, the spatial distribution of the overflow charges at the end of laser irradiation is shown in Figure 8c. At the time the readout transfer occurs, the transmission potential wells carrying the overflow charges have moved to the downstream pixels at positions No. 260 to No. 520. These overflow charges will be considered as attributing to the downstream pixels at positions No. 260 to No. 520. Therefore, the transmission potential wells that acquire the overflow charges before the readout transfer will affect the downstream pixels. However, the overflow charges acquired by the transfer potential well passing through the overflow pixel after the readout transfer will be considered as attributing to the upstream pixels at positions No. 520 to No. 780 to which it is bound. Therefore, the transport potential wells that acquire the overflow charges after the readout transfer will affect the upstream pixel. Eventually, when the laser irradiation ends, the transmission potential wells No. 260 to No. 780 acquire the overflow charges, as shown in Figure 8c, resulting in the output image of the CCD, as shown in Figure 6c. When Δ*t* is 32 ms, the spatial distribution of the overflow charges at the end of the laser irradiation is shown in Figure 8d. At the end of the laser irradiation, the transmission potential wells from No. 520 to No. 1040 all acquire overflow charges. These transmission potential wells are bound to the upstream pixels at positions No. 520 to No. 1040 when the previous readout transfer action occurs. Eventually, these overflow charges will be considered as signal charges obtained from the upstream pixels at positions No. 520 to No. 1040, resulting in the output image of the CCD, as shown in Figure 6d.

### 3.2. CCD Irradiating by Continuous Laser in Motion Mode

#### 3.2.1. Effect of Rotation Speed

**Figure 9:**
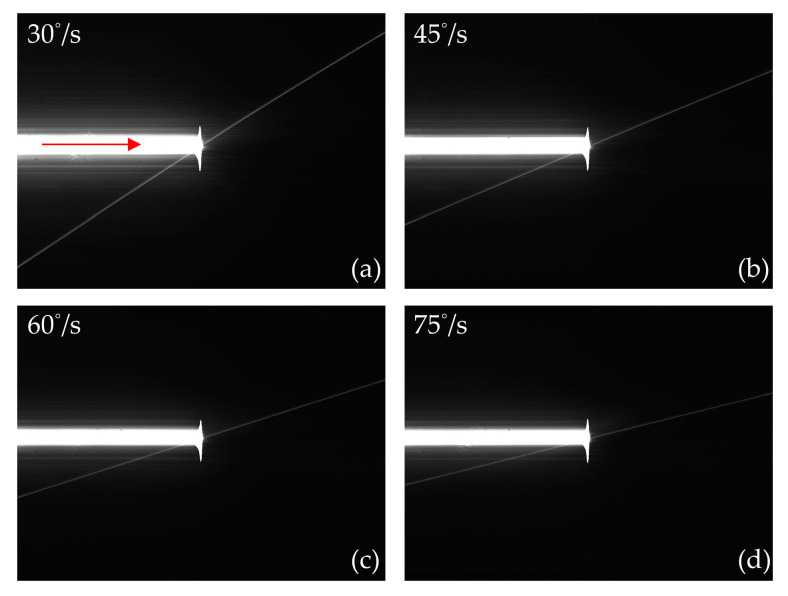
The output images of the CCD produced by scanning the CCD with a continuous laser at different rotational speeds of the motorized rotation stage. (**a**) The rotational speed of the motorized rotation stage is 30°/s; (**b**) The rotational speed of the motorized rotation stage is 45°/s; (**c**) The rotational speed of the motorized rotation stage is 60°/s; (**d**) The rotational speed is 75°/s. The scanning direction of the continuous laser is shown by the red arrow.

Figure 9 shows the output images produced by scanning the CCD with a continuous laser at different rotational speeds of the motorized rotation stage. The output images of the CCD in Figure 9 mainly contain rectangular-shaped laser spots and crosstalk lines with a certain slope. In addition, a longitudinal short line with a saturated gray value appears at the right end of the rectangular light spot. The reason is that the signal charges overflow from the transmission potential well in the vertical register. When the read transfer occurs, all the charges in the collecting potential wells are transferred to the transmission potential wells that have obtained the overflow charges. Therefore, these transmission potential wells reach the maximum capacity and spill the charges into the adjacent potential well in the vertical register. In addition, compared with the experimental results obtained under static conditions, the grid distribution of light points and the divergence of light distributed around the laser spot cannot be observed in the output images, which is caused by the change in the direction of the optical axis during rotation. During the initial stage of laser scanning, all irradiated pixels generate signal charges that can be transferred to the transmission potential well by the readout transfer. Furthermore, the readout transfer occurs when the laser scans into the middle of the CCD. The signal charges generated by the pixels scanned by the laser could not be transferred to the transmission potential well of the vertical register by the readout transfer, resulting in a rectangular saturated laser spot distributed only in the left half of the output image. In addition, the formation of crosstalk lines with a certain slope is related to the horizontal scanning of the laser. As the laser spot is always moving, the charges overflow sequentially into each of the vertical registers that the laser has scanned rather than overflowing into only a few neighboring vertical registers. Moreover, the transmission potential wells in each vertical register are continuously moving downward during the laser scanning process. Therefore, the crosstalk line with a certain slope is eventually formed. In addition, the laser scanning speed has an important influence on the slope of the crosstalk line. The slope *k* of the crosstalk line can be calculated by the following equation:

(1) $$k=\frac{\Delta y}{\Delta x}=\frac{v_{y}\cdot T_{L}}{v_{x}\cdot T_{L}}=\frac{v_{y}}{v_{x}},$$

where Δ*y* and Δ*x* are the increments in the number of pixels on the crosstalk line in the *y* and *x* directions, respectively. *v_x_* and *v_y_* are the laser scanning speed and the motion speed of the transmission potential well in the vertical register, respectively. *T_L_* is the time required for the laser to complete the scanning of the CCD. When the integration time of the CCD is *T_e_*, the moving speed *v_y_* of the vertical transmission potential well can be calculated by the following equation:

(2) $$v_{y}==\frac{H}{T_{e}},$$

where *H* is the total number of pixels in the vertical direction of the CCD chip. When the integration time is constant, *v_y_* is a constant. Therefore, *k* is inversely proportional to *v_x_*. As *v_x_* is proportional to the rotational speed of the motorized rotation stage, the slope of the crosstalk line gradually decreases as the rotational speed increases from 30°/s to 75°/s.

#### 3.2.2. Effect of Positions Scanned by Moving Laser

**Figure 10:**
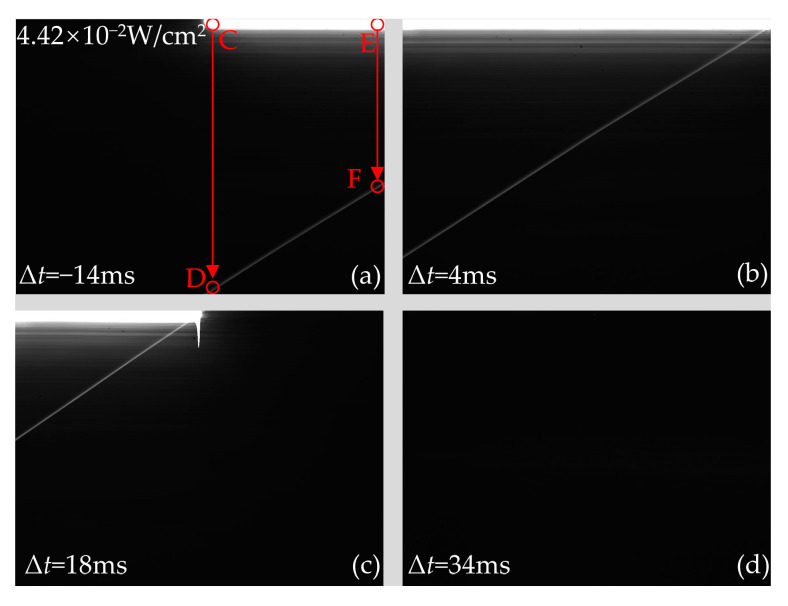
The output images of the CCD produced by laser continuous at the laser fluence of 4.42 × 10^−2^ W/cm^2^ when the continuous laser scans the top of the CCD with typical delay times of (**a**) Δ*t* = −14 ms, (**b**) Δ*t* = 4 ms, (**c**) Δ*t* = 18 ms, and (**d**) Δ*t* = 34 ms, respectively. The rotational speed of the motorized rotation stage is 30°/s.

**Figure 11:**
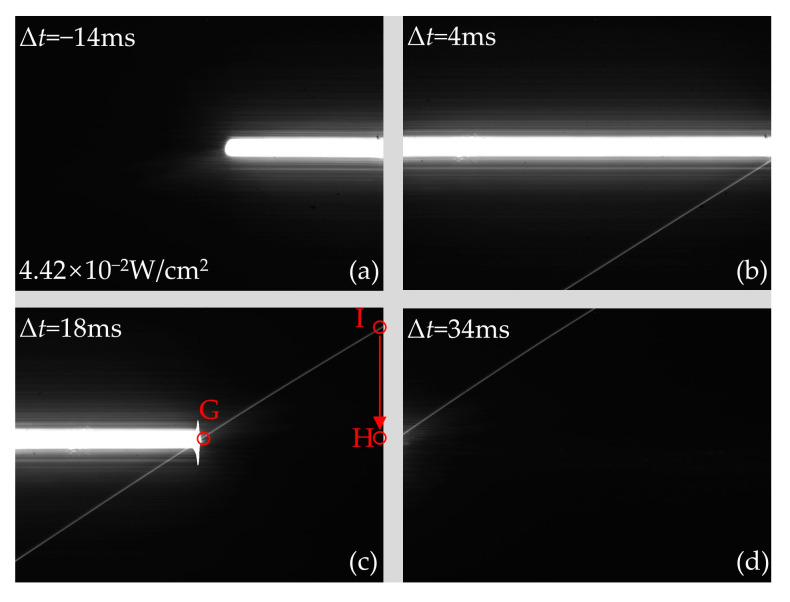
The output images of CCD produced by laser continuous at the laser fluence of 4.42 × 10^−2^ W/cm^2^ when the continuous laser scans the middle of the CCD with typical delay times of (**a**) Δ*t* = −14 ms, (**b**) Δ*t* = 4 ms, (**c**) Δ*t* = 18 ms, and (**d**) Δ*t* = 34 ms, respectively. The rotational speed of the motorized rotation stage is 30°/s.

**Figure 12:**
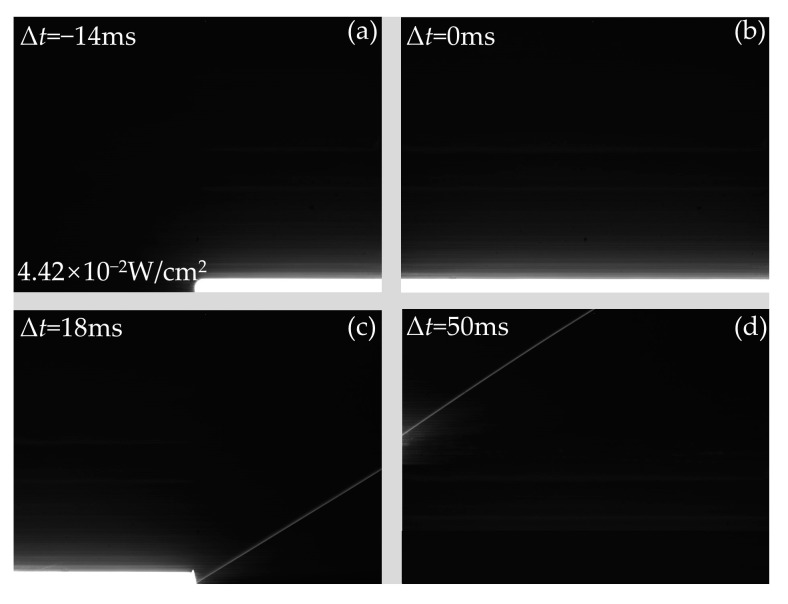
The output images of the CCD produced by laser continuous at the laser fluence of 4.42 × 10^−2^ W/cm^2^ when the continuous laser scans the bottom of the CCD with typical delay times of (**a**) Δ*t* = −14 ms, (**b**) Δ*t* = 0 ms, (**c**) Δ*t* = 18 ms, and (**d**) Δ*t* = 50 ms, respectively. The rotational speed of the motorized rotation stage is 30°/s.

When the laser fluence is 4.42 × 10^−2^ W/cm^2^, the motorized rotation stage drives the laser to scan the CCD from left to right at a speed of 30°/s. The top, middle, and bottom of the CCD were selected as the typical irradiation positions, and the CCD output images with different delay times are shown in Figure 10, Figure 11, and Figure 12, respectively. When the rotational speed is 30°/s, according to Equation (1) and the slope of the crosstalk line in Figure 9a, the scanning speed of the continuous laser *v_x_* can be calculated to be 50 pixel/ms or 0.3225 m/s. Therefore, the time *T_L_* required for the continuous laser to scan the CCD in the horizontal direction can be obtained as follows:

(3) $$T_{L}=\frac{L}{v_{x}}=28\;\mathrm{ms}$$

where *L* is the total number of pixels in the horizontal direction of the CCD chip.

When the continuous laser scans the top of the CCD, the output images with typical delay times are shown in Figure 10. As shown in Figure 10, the delay time has an important effect on the distribution of crosstalk lines and laser spots. When the delay time Δ*t* is −14 ms, the rectangular laser spot is distributed in the right half of the output image. This is because the CCD does not start integrating until the laser reaches the middle of the CCD. When Δ*t* is 4 ms, the laser spot traverses the entire CCD. The reason is that it takes only 28 ms for the laser to complete scanning the CCD, which is shorter than the integration time of 32 ms. Therefore, the CCD is scanned by the laser before the readout transfer, causing the laser spot to cross the CCD. When the Δ*t* is 18 ms, the laser spot is mainly distributed in the left half of the output image. This is because the readout transfer occurs when the laser scans into the middle of the CCD. Since there is only one action of readout transfer in one experiment, the signal charges generated by laser scanning the right half of the CCD cannot be read out, resulting in the laser spot only distributed in the left half of the output image. In addition, signal charges will overflow in the pixels scanned by the laser due to the intense laser fluence. As shown in Figure 10a, when the laser passes through point C, the overflow charges will move downward with the transmission potential well, owing to the filling of the collecting potential well with photogenerated charge. When the readout transfer occurs, the transmission potential well carrying the overflow charges moves to point D. These overflow charges will be considered as the signal charges generated by the pixel at point D. As a result, the gray value of the pixel at point D in the CCD output image is relatively large. When the laser scans to point E, the transmission potential well obtains overflow charges at point E. Subsequently, the transmitting potential well carrying the overflow charges arrives at point F when the readout transfer occurs. Eventually, when the laser scans from point C to point E, a crosstalk line with a certain slope is formed between points D and F. However, as shown in Figure 10d, the laser has no interference effect on the CCD when the delay time Δ*t* is 34 ms. This is because the image acquisition cycle of the CCD has ended.

When the continuous laser scans the middle of the CCD, the CCD output images obtained at typical delay times are shown in Figure 11. When the delay time is the same, the distribution of the rectangular-shaped laser spot in the horizontal direction in Figure 11 is the same as that in Figure 10. However, the distribution of the crosstalk lines in Figure 11 is significantly different from that in Figure 10. As shown in Figure 11a, the crosstalk line cannot be observed in the output image of the CCD. The reason is that overflow charges carried by the transmission potential wells have already left the vertical register before the readout transfer occurs. These overflow charges will not affect the pixels, resulting in no crosstalk line, as shown in Figure 11a. In addition, crosstalk lines appear above the laser spot in Figure 11c,d. This is due to the occurrence of the readout transfer when the laser is scanned to point G. The transmission potential wells bound to the pixels between points G, and I sequentially receive overflow charges while passing through the pixels between points G and H. The overflow charges will be considered as the signal charges generated from the pixels between points G and I. As a result, the crosstalk line between points G and I is formed.

When the continuous laser scans the bottom of the CCD from left to right, the output images at typical delay times are shown in Figure 12. As shown in Figure 11 and Figure 12, when the delay time is the same, the distribution of the laser spot in the horizontal direction is the same. However, the distribution of the laser spot in the vertical direction is different due to the different positions of laser irradiation. Because the irradiation position is at the bottom of the CCD, the crosstalk lines in the output image are distributed only above the laser spot. In addition, by comparing Figure 11d and Figure 12d, the distribution of crosstalk lines in the output image of the CCD is the same, while the delay time is different by 16 s due to the different positions of the laser irradiation.

**Figure 13:**
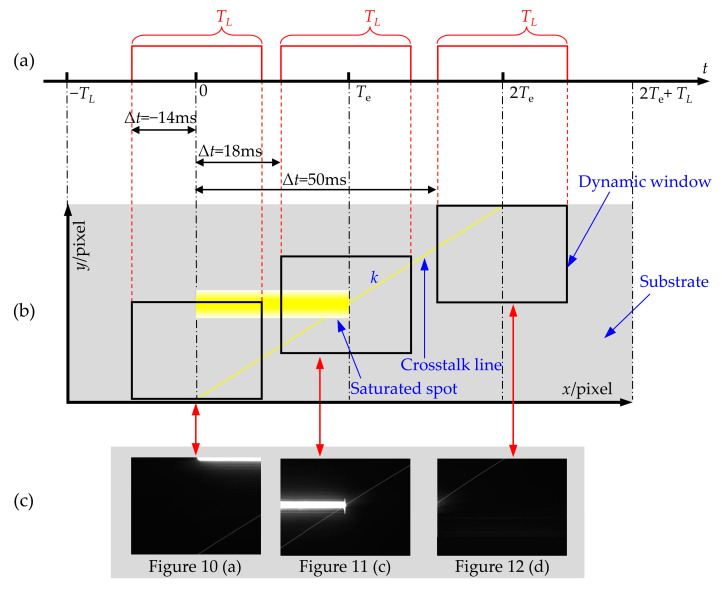
(**a**) Timing schematic diagram of CCD working process and laser scanning; (**b**) The generic law graph of the spatial and temporal distribution of the laser spot and crosstalk line when the CCD is scanned by the continuous laser; (**c**) Typical experimental results of the output images of the CCD.

Based on the conclusion of the spatial distribution of laser spots and crosstalk lines in Figure 10, Figure 11 and Figure 12 at different delay times, the generic law of the spatial and temporal distribution of the laser spot and crosstalk line is shown by the graph in Figure 13. The three square wave signal in Figure 13a is the diagram of the working time sequence when the continuous laser scans the top, middle, and bottom of the CCD at typical delay times, respectively. The rising and falling edges of the square wave correspond to the beginning and end of the scanning CCD by the continuous laser, respectively. *T_L_* is the time required for the continuous laser to finish scanning the CCD. In addition, the time 0 and time *T_e_* in Figure 13a are the start of the integration of the CCD and the time of readout transfer, respectively. Figure 13b mainly shows the crosstalk line, saturated laser spot, and dynamic window. As shown in Figure 13b, the rectangular-shaped laser spot is in the middle of the substrate in the *y*-direction and between time 0 and time *T_e_* in the *x*-direction. In addition, the slope of the crosstalk line is calculated by Equations (1) and (2). The crosstalk line passes through the right endpoint of the rectangular laser spot. The width of the dynamic window corresponds to the width of the square wave signal in Figure 13a, and the aspect ratio of the dynamic window is the same as that of the CCD chip. The position of the dynamic window depends on the irradiation position and the delay time. When the position of the dynamic window is determined, the spatial distribution of crosstalk lines in the dynamic window is the theoretical output image of the CCD. The experimental results of the typical output images are shown in Figure 13c. By comparing the theoretical output images in the dynamic window in Figure 13b with the experimental results in Figure 13c, the spatial distribution of the crosstalk lines in the output image of the CCD is better predicted.

## 4. Conclusions

The output images of the CCD produced by irradiating a progressive scan CCD with a continuous laser in static mode were experimentally recorded. The threshold for the appearance of the crosstalk line is 1.59 × 10^−3^ W/cm^2^. In addition, the grey values of the crosstalk line are divided into two stages by the laser fluence of 8.88 × 10^−2^ W/cm^2^: linear increase and saturation, which correspond to different formation mechanisms of the crosstalk lines, respectively. When the duration of laser irradiation is less than the integration time of the CCD, the spatial distribution of the laser spots and crosstalk lines at different delay times is experimentally observed. The laser irradiation before the readout transfer will cause the saturated laser spot, and the crosstalk line below the laser spot to be observed in the output image of the CCD. Moreover, the laser irradiation after the readout transfer will cause the crosstalk line above the irradiation position to appear in the output image.

When the continuous laser scans the CCD from left to right at different speeds, the output images of the CCD mainly consist of rectangular-shaped laser spots and crosstalk lines with certain slopes. In addition, the slope of the crosstalk line is determined by the movement speed of the transmission potential well in the vertical register and the speed of laser scanning. In addition, the delay time and the irradiation position of the continuous laser have an important effect on the spatial distribution of the crosstalk line. This study provides a technical reference for the laser protection and structure optimization design of a CCD.

## Data Availability

Data are contained in the article.

## References

[1] Ramazanov A., Simon V., Uhov A., Kostrin D., Gerasimov V., Selivanov L. (2017). Modification of the CCD Photodetectors for the Suppression of Interference in Their Internal Structure. Proceedings of the International Conference PhysicA.SPb/2017.

[2] Towaranonte B., Gao Y. (2022). Application of Charge-Coupled Device (CCD) Cameras in Electrochemiluminescence: A Minireview. Anal. Lett..

[3] Baranov M. (2020). Image Processing of Biological Liquids Films for Medical Diagnostics. J. Electron. Sci. Technol..

[4] Choi E., Choi S., An K., Kang K. (2024). Deep Learning-Based Inkjet Droplet Detection for Jetting Characterizations and Multijet Synchronization. ACS Appl. Mater. Interfaces.

[5] Oh S., Kang H. (2017). Object Detection and Classification by Decision-Level Fusion for Intelligent Vehicle Systems. Sensors.

[6] Li Y., Kou Z., Wang G., Hou Y., Xie X., Yu Y., Wang Y., Lu Z. (2022). Recent Advances of Short-Pulse Laser–Induced Breakdown Effect on Charge-Coupled Device Detectors. Opt. Laser Technol..

[7] Li G., Shen H., Li L., Zhang C., Mao S., Wang Y. (2013). Laser-Induced Damages to Charge Coupled Device Detector Using a High-Repetition-Rate and High-Peak-Power Laser. Opt. Laser Technol..

[8] Gopinath D., Soman M., Holland A., Keelan J., Hall D., Holland K., Colebrook D. (2018). Soft X-ray Radiation Damage in EM-CCDs Used for Resonant Inelastic X-ray Scattering. J. Instrum..

[9] He B., Yao Z., Zhang F. (2009). A Comparison of Ionizing Radiation Damage in CMOS Devices from 60Co Gamma Rays, Electrons and Protons. Chin. Phys. C.

[10] Zhang Z., Cheng X., Jiang T., Jiang Z. (2012). A Dazzling Phenomenon of CW Laser on Linear CCD Camera. Optik.

[11] Kou Z., Li Y., Wang G., Li K., Hou Y., Xie X., Yu Y., Wang Y., Lu Z. (2022). Temporal Evolution Characteristics and Mechanism Analysis of CCD Breakdown Induced by Nanosecond and Picosecond Pulse Lasers. Optik.

[12] Zhang Z., Shi Y., Li Y., Dou P., Xu Z., Zhang J. Performance Jump Characteristics of CCD Image Sensor under Laser Irradiation. Proceedings of the Fifth International Symposium on Laser Interaction with Matter.

[13] Zhang Z., Cheng X., Wang R., Jiang T., Qiu D., Jiang Z. (2011). Dazzling Effect of Repetitive Short Pulse Laser on TDI CCD Camera. Opt. Lasers Eng..

[14] Sun K., Huang L., Cheng X., Jiang H. (2011). Analysis and Simulation of the Phenomenon of Secondary Spots of the TDI CCD Camera Irradiated by CW Laser. Opt. Express.

[15] Hou J., Lu Q., Shu B. Saturation of Charge-Coupled Devices Irradiated by Laser out of the Field of View. Proceedings of the International Conference on Industrial Lasers.

[16] Zhang Y., Niu C., Zhao S., Lv Y. (2020). Investigation of Interference on Photodetector CCD by Lasers with Different Wavelengths. Optik.

[17] Tang Y., Yue M., Zhang J., Fang X., Feng X. (2020). Removal of Optical Crosstalk Caused by Light Source for Synchronous Measurement of Temperature and Deformation. Opt. Eng..

[18] Zhang Z., Zhou M., Cheng D., Zhang J. (2015). Analysis and Simulation of Entirely Saturated Unilateral Laser Spot Tails in BCCD. Opt. Express.

[19] Zhang Z., Cheng D., Shi Y., Zhang J. (2017). The Terminal Vibration of Laser Spot Tail in Dual Channel Type Linear CCD. Opt. Laser Technol..

[20] Zhang Z., Cai Y., Zhang J., Wei C., Feng G., Ye X. Analysis and Simulation to Excessive Saturation Effect of CCD. Proceedings of the 2nd International Symposium on Laser Interaction with Matter (LIMIS 2012).

[21] Xu J., Zhao S., Hou R., Li X., Wu J., Li Y., Meng W., Ni Y., Ma L. (2009). Laser-Jamming Analysis of Combined Fiber Lasers to Imaging CCD. Opt. Lasers Eng..

[22] Zhang Z., Zhang J., Shao B., Cheng D., Ye X., Feng G. Typical Effects of Laser Dazzling CCD Camera. Proceedings of the Third International Symposium on Laser Interaction with Matter (LIMIS 2014).

[23] Wang X., Nie J., Li H., Lei P., Hao X. (2013). Experiment Research on 1064nm Laser of High Pulse-Repetition-Frequency Disturbing Visible CCD Detectors. Infrared Laser Eng..

[24] Wang Y., Chen Q., Xu G., Ren G., Zhou X., Li H., Zhu R. The Influence of Detector’s Integration Time on the Laser Disturbing Effect. Proceedings of the 14th National Conference on Laser Technology and Optoelectronics (LTO 2019).

[25] Wang Y., Wang G., Chen Q., Li H., Zou Q., Wang M. (2015). Influence of Detector’s Gain on Laser Disturbing Effect. Chin. J. Lasers.

[26] Wen J., Bian J., Li X., Kong H., Guo L., Lv G. (2023). Research Progress of Laser Dazzle and Damage CMOS Image Sensor. Infrared Laser Eng..

[27] Guo J., Wang T. (2020). Mechanism and Assessment of Laser Irradiation of Opto-Electrical Systems.

[28] Li M., Jin G., Tan Y., Guo M., Zhu P. (2016). Study on the Mechanism of a Charge-Coupled Device Detector Irradiated by Millisecond Pulse Laser under Functional Loss. Appl. Opt..

[29] Wang Y., Liu Y., Chen Q., Li H., Zhou X., Ren G., Zhu R. (2018). Mesh Distribution of Laser Energy on the Photosensitive Surface of CCD. Infrared Laser Eng..

